# Navigating freely-available software tools for metabolomics analysis

**DOI:** 10.1007/s11306-017-1242-7

**Published:** 2017-08-09

**Authors:** Rachel Spicer, Reza M. Salek, Pablo Moreno, Daniel Cañueto, Christoph Steinbeck

**Affiliations:** 1 European Molecular Biology Laboratory, European Bioinformatics Institute (EMBL-EBI), Wellcome Trust Genome Campus, Hinxton, Cambridge, CB10 1SD UK; 2 Friedrich-Schiller-University Jena, Lessingstr. 8, Jena, 07743 Germany; 3 Metabolomics Platform, IISPV, DEEEA, Universitat Rovira i Virgili, Campus Sescelades, Carretera de Valls, s/n, 43007 Tarragona, Catalonia Spain

**Keywords:** Metabolomics, Bioinformatics, Software, Freely available, Data analysis

## Abstract

**Introduction:**

The field of metabolomics has expanded greatly over the past two decades, both as an experimental science with applications in many areas, as well as in regards to data standards and bioinformatics software tools. The diversity of experimental designs and instrumental technologies used for metabolomics has led to the need for distinct data analysis methods and the development of many software tools.

**Objectives:**

To compile a comprehensive list of the most widely used freely available software and tools that are used primarily in metabolomics.

**Methods:**

The most widely used tools were selected for inclusion in the review by either ≥ 50 citations on Web of Science (as of 08/09/16) or the use of the tool being reported in the recent Metabolomics Society survey. Tools were then categorised by the type of instrumental data (i.e. LC–MS, GC–MS or NMR) and the functionality (i.e. pre- and post-processing, statistical analysis, workflow and other functions) they are designed for.

**Results:**

A comprehensive list of the most used tools was compiled. Each tool is discussed within the context of its application domain and in relation to comparable tools of the same domain. An extended list including additional tools is available at https://github.com/RASpicer/MetabolomicsTools which is classified and searchable via a simple controlled vocabulary.

**Conclusion:**

This review presents the most widely used tools for metabolomics analysis, categorised based on their main functionality. As future work, we suggest a direct comparison of tools’ abilities to perform specific data analysis tasks e.g. peak picking.

**Electronic supplementary material:**

The online version of this article (doi:10.1007/s11306-017-1242-7) contains supplementary material, which is available to authorized users.

## Introduction

Metabolomics has been described as the study of the entirety of the endogenous small molecules present within an organism, organ, biological tissue or cell (Fiehn 2002). After the first occurrences of the term in 1998, the field has dynamically grown over the past two decades and is now maturing (Kell and Oliver 2016). Due to the diversity of classes of metabolites, a number of different analytical chemistry techniques are required to sample this physicochemical space, since no single analytical technique alone is able to capture the entire metabolome. Instead different, often complementary, techniques are used to measure specific portions of the metabolome. The three most frequently used technologies are: liquid chromatography–mass spectrometry (LC–MS), gas chromatography–mass spectrometry (GC–MS) and nuclear magnetic resonance (NMR). Some alternative and less commonly used platforms include direct injection (DI) and capillary electrophoresis- (CE-) mass spectrometry, diode-array detector, infrared and RAMAN spectroscopy. The data produced from each analytical method requires distinct handling and thus different data analysis tools and workflows are required.

The majority of metabolomics practitioners’ day-to-day activities now consists of a combination of wet and dry lab work and only half have dedicated bioinformatics support (Weber et al. 2016). It is therefore important that users are made aware of the range of tools available for data analysis. Tools should also be intuitive, user-friendly and ideally open source.

There are, of course, also disadvantage to using open source software (Earll 2012). It can contain bugs, as any kind of software. Due to the software being open source, however, bugs are shallow and can be fixed outside of the potentially nontransparent release cycles of close source software. Old open source software may not be maintained. Closed source commercial software can have the advantage of ease-of-use, being well tested and documented, and can be tailored to individual users. It too, however, also has disadvantages. Unlike open source software, algorithms are kept in a “black box” and there is a lack of transparency to precisely how analysis is performed. Commercial software can also be prohibitively expensive.

This review will therefore focus on tools that are freely available to use: open-source, free for non-commercial or free to use. Whilst tools written in MATLAB (MathWorks) and Mathematica (Wolfram Mathematica: Modern Technical Computing) may be freely available to use, tools written in these languages will not be focused upon. The open source GNU Octave may run MATLAB based software, however testing this is beyond the scope of this review.

In this review software are classified into the following categories based on their major functionality: Preprocessing, Annotation, Post-processing, Statistical analysis, Workflows and Other tools. Tools designed for Preprocessing and Annotation will also be further subdivided by the instrumental data type they are designed for the analysis of. Preprocessing software is split into LC–MS, GC–MS and NMR. Annotation tools are separated into mass spectrometry and NMR, with NMR also including quantification. Mass spectrometry annotation tools are partitioned by level of annotation provided into: Level 4: unequivocal molecular formula, Level 3: tentative candidates and Level 2a: library spectrum match. These classifications are based on criteria by Schymanski et al. (2014). Software that provide Preprocessing, Annotation and Statistical analysis are classified as Workflows. Tools whose main purpose does not fit into any other class are included in the Other tools category. Some of the tools mentioned may also have other uses that are not mentioned in the text of this review.

Despite accounting for an important section of data analysis, tools for pathway analysis will not be included in this review. This is because the majority of tools in this area are not designed specifically for metabolomics. For an overview of methods and software tools for pathway analysis see Booth et al.’s (2013) review.

With journals and funding bodies increasing requiring data, data deposition is an important final stage of metabolomics data handling. The ISA software suite (Rocca-Serra et al. 2010) provides tools for experimental metadata management. For depositing data to the MetaboLights (Haug et al. 2013) repository users must submit experimental metadata in the ISA-Tab format. Conversely, users submitting metadata to the Metabolomics Workbench (Sud et al. 2016) repository must complete an online form or a supplied excel template.

As there is a large number of software specifically designed for metabolomics data analysis, ~200, only the most widely used tools will be included in the text of this review. The criteria for inclusion for being considered ‘the most widely used’ for the purpose of this review is either ≥50 citations on Web of Science (as of 08/09/16) or the use of the tool being reported in the recent Metabolomics Society survey (Weber et al. 2016). Compared to other previous reviews of metabolomics software tools (Misra and van der Hooft 2016; Sugimoto et al. 2012), this review aims to supply a greater amount of information about each tool included. Whilst including extra information is not possible in the body of the review, a more detailed list of tools is included in Supplementary Table 1. Additional information about each tool includes accepted data input formats, programming language written in, dependencies and dates of publication and most recent update. As far as the authors are aware, no earlier reviews of metabolomics software include such extensive information about the included tools. All information in the supplementary material is further available at https://github.com/RASpicer/MetabolomicsTools. The GitHub wiki also further includes tools written in MATLAB and Mathematica and tools designed for pathway analysis.

## Preprocessing

The majority of freely available software tools for preprocessing require MS data to be in an open format e.g. mzML, mzXML and netCDF, although some will also accept raw data in proprietary formats (see Supplementary Table 1). The first stage before preprocessing is thus often conversion to an open data format. The majority of vendor software that comes shipped with instruments provides the option of converting data to the netCDF format (Rew and Davis 1990). The proteowizard (Chambers et al. 2012) project tool msconvert converts from most proprietary formats to mzML (Turewicz and Deutsch 2010) and mzXML (Pedrioli et al. 2004). When possible it is recommended that the mzML format be used, as it uses zlib compression to produce smaller file sizes (Martens et al. 2011) compared to mzXML and mzData, and it is still under active development, with new technologies being incorporated. However, there are still more tools that will accept the mzXML and netCDF formats as input, as they are older file formats.

The initial stages of data preprocessing are similar for LC–MS and GC–MS metabolomics. Typically the pipeline consists of peak picking, deconvolution, peak matching and peak alignment across samples (Want and Masson 2011). The first stage of peak detection can also consist of baseline correction, noise reduction and smoothing, depending on the algorithm used. Deconvolution is necessary for handling overlapping peaks and fragments originating from the same metabolite. Prior to alignment, peaks are matched/grouped by m/z and retention time. Routinely peak alignment is performed using retention times for LC–MS (Zhou et al. 2012). For GC–MS retention times are generally converted into instrument independent retention indices (RI), for comparison to existing databases for compound identification (Chen et al. 2011), although alignment techniques that do not require RI also exist (Domingo-Almenara et al. 2016). GCxGC-MS is also becoming an increasingly used analytical technique and specific software for the preprocessing of GC × GC–MS data is required (Winnike et al. 2015).

In NMR metabolomics, signals are generated as free induction decay (FID). The spectra must be transformed from FID collected in time domain into frequency spectra prior to any subsequent analysis (Ellinger et al. 2013). This means that the preprocessing of NMR metabolomics data differs from MS, with the first stages consisting of zero-filling, apodization, Fourier transformation and phase correction (Morris 2017; Ren et al. 2015; Smolinska et al. 2012; Vettukattil 2015). The other later stages of baseline correction, deconvolution, binning, peak alignment, scaling and normalisation are same as for MS, although the precise algorithms used may vary.

Because of the different nature of LC-MS, GC-MS and NMR preprocessing workflows, this section is split into three subsections: LC–MS, GC–MS and NMR. Every tool referenced in these sections is also included in Table 1. A further 41 tools for preprocessing are included in Supplementary Table 1.

**Table 1:** Software tools commonly used for the preprocessing of metabolomics data

Tool	Instrument data type	Software type	Website	References
XCMS	LC–MS, GC–MS	R Package	http://bioconductor.org/packages/release/bioc/html/xcms.html	Smith et al. (2006)
OpenMS—FeatureFinderMetabo	LC–MS	GUI	http://ftp.mi.fu-berlin.de/pub/OpenMS/release-documentation/html/TOPP_FeatureFinderMetabo.html	Bertsch et al. (2010)
MetAlign	LC–MS	Windows GUI	http://www.wageningenur.nl/en/show/MetAlign-1.htm	Lommen & Kools (2012)
MS-DIAL	LC–MS	Windows GUI	http://prime.psc.riken.jp/Metabolomics_Software/MS-DIAL/index.html	Tsugawa et al. (2015)
mzMatch	LC–MS	R Package	http://mzmatch.sourceforge.net/index.php	Scheltema et al. (2011)
IDEOM	LC–MS	Excel Template	http://mzmatch.sourceforge.net/ideom.php	Creek et al. (2012)
AMDIS	GC–MS	Windows GUI	http://chemdata.nist.gov/dokuwiki/doku.php?id=chemdata:amdis	Meyer et al. (2010)
MetaboliteDetector	GC–MS	CLI, GUI	http://md.tu-bs.de	Hiller et al. (2009)
MET-IDEA	GC–MS	Windows CLI	http://bioinfo.noble.org/download	Broeckling et al. (2006)
MeltDB	LC–MS, GC–MS	Web App	https://meltdb.cebitec.uni-bielefeld.de/cgi-bin/login.cgi	Kessler et al. (2013)
metaMS	GC–MS	R Package	http://bioconductor.org/packages/release/bioc/html/metaMS.html	Wehrens et al. (2014)
MSeasy	GC–MS	R Package	https://cran.r-project.org/web/packages/MSeasy/index.html	Nicolè et al. (2012)
SpectConnect	GC–MS	Web App	http://spectconnect.mit.edu	Styczynski et al. (2007)
rNMR	NMR	R Package	http://rnmr.nmrfam.wisc.edu	Lewis et al. (2009)

*CLI* command line interface, *GUI* graphical user interface

### LC–MS preprocessing

Many of the established preprocessing tools for LC–MS data are implemented as R packages, including XCMS (Smith et al. 2006), the most used software for LC–MS analysis, as reported in a recent survey, with 70% of respondents reporting to use it (Weber et al. 2016). Recent updates to these tools mean data analysis from a wider variety of experimental conditions and technologies is now supported. Unsurprisingly, since LC–MS is the most widely used analytical technique in metabolomics, a far greater number of software has been developed for preprocessing LC–MS metabolomics data than for GC–MS or NMR.

XCMS now contains 7 different peak detection algorithms (Smith et al. 2006; Du et al. 2006; Treutler and Neumann 2016), including Massifquant (Conley et al. 2014), as well as the established matchFilter (Smith et al. 2006) and CentWave (Tautenhahn et al. 2008) methods. The Massifquant (Conley et al. 2014) algorithm is an open-source implementation of the TracMass (Döös et al. 2013) algorithm that is designed for isotope trace detection. There are also three methods provided for peak grouping and two for retention time alignment: loess and obiwarp (ordered bijective interpolated warping) (Prince and Marcotte 2006).

A growing number of users are adopting a workflow based approach for their LC–MS data processing, for example XCMS Online (Tautenhahn et al. 2012), Metabolomic Analysis and Visualization ENgine (MAVEN) (Melamud et al. 2010) MZmine2 (Pluskal et al. 2010), MetaboAnalyst (Xia et al. 2015) and metabolomics specific Galaxy workflows—Galaxy-M (Davidson et al. 2016) and Workflow4metabolomics (Giacomoni et al. 2014). More detail on these tools will be included in the later section—Workflows.

Other freely available software reported in the survey (Weber et al. 2016), which are more specifically designed for data preprocessing were OpenMS (Bertsch et al. 2010), MetAlign (Lommen and Kools 2012) and Mass Spectrometry-Data Independent AnaLysis (MS-DIAL) (Tsugawa et al. 2015). OpenMS (Bertsch et al. 2010) is a library for LC–MS data analysis. It was originally designed for proteomics, however it now also includes the FeatureFinderMetabo (Kenar et al. 2014) module, specifically designed for non-targeted metabolomics data. It incorporates peak picking, noise filtering, retention time (RT) alignment, metabolite quantification and identification. Isotopes are identified using a HiRes (Zhao et al. 2006) generated library. A number of preprocessing functions are provided by MetAlign (Lommen and Kools 2012) including peak-picking, retention time alignment, noise reduction, baseline correction and missing value filling. MS-DIAL (Tsugawa et al. 2015) provides deconvolution of untargeted data-independent acquisition (DIA) MS/MS data using the MS2Dec algorithm. An algorithm based on the Joint Aligner from MZmine (Pluskal et al. 2010) is used for peak alignment.

mzMatch (Scheltema et al. 2011) provides data preprocessing of LC–MS data, based upon the PeakML file format. It also includes isotopic labelling analysis (Chokkathukalam et al. 2013) and probabilistic metabolite annotation (Daly et al. 2014). IDEOM (Creek et al. 2012) is an excel template, which provides a GUI with implementations of mzMatch and XCMS, along with macros for noise-filtering, metabolite identification and statistical analysis. It can also interface directly with msconvert (Chambers et al. 2012), which converts MS data from vendor formats into the open .mzML and .mzXML formats.

### GC–MS preprocessing

Despite the relative ease of feature annotation, GC–MS is a less used analytical technique for metabolomics than LC–MS, as it is only able to detect volatile and thermally stable compounds and those that can be rendered volatile by chemical derivatization. This means that compared to LC–MS, far less analytes can be detected. However, the advantage of GC–MS is that it is a more robust and reproducible analytical technique with established libraries and databases for metabolite identification.

The long-standing AMDIS (Meyer et al. 2010) (Automated Mass Spectral Deconvolution and Identification System) is the most widely used freely available tool for GC–MS data processing. Whilst it was originally designed for the automatic identification of chemical weapons it is applicable to all GC–MS data, including metabolomics. Spectra are deconvoluted to extract pure compound peaks free of overlapping signals from the total ion chromatograms. Pure compound peaks are then matched to a user-defined target library, using the additional parameters of peak shape and retention time. Importantly AMDIS does not include spectral alignment, so other software must additionally be used.

Surprisingly XCMS (Smith et al. 2006) was second most widely used open source software for GC–MS analysis in the Metabolomics Society survey (Weber et al. 2016), despite being primarily designed for LC–MS analysis and having no functions specifically for GC–MS analysis.

Gas chromatography–Mass spectrometry specific preprocessing software MetaboliteDetector (Hiller et al. 2009), MET-IDEA (Broeckling et al. 2006), MeltDB (Kessler et al. 2013), metaMS (Wehrens et al. 2014) and MSeasy (Nicolè et al. 2012) were also reported to be used (Weber et al. 2016). MetaboliteDetector (Hiller et al. 2009) incorporating baseline correction, smoothing, peak detection and deconvolution. In Niu et al.’s (2014) comparison of peak detection software it scored highly in both trials of true peak detection, coming 1st and 2nd respectively. Surprisingly, whilst metAlign (Lommen and Kools 2012) is designed for the analysis of LC–MS data, it also performed well in the same trial. MET-IDEA is designed to take AMDIS (Meyer et al. 2010) output as input and can quantify the results. It generates a list of mass spectral tags from the inputted ion list. A suite of modular tools is provided by MeltDB. It includes a number of algorithms for peak picking including matchFilter (Smith et al. 2006) and centWave (Tautenhahn et al. 2008) from XCMS and MassSpecWavelet (Du et al. 2006), as well as retention indices calculation and sum formula annotation.

metaMS(Wehrens et al. 2014) is based on XCMS (Smith et al. 2006) and CAMERA (Kuhl et al. 2012) but is adapted for GC–MS analysis. Unlike XCMS, metaMS performs pseudospectra analysis, avoiding the alignment stage that can be difficult to execute with GC-MS. In MSeasy (Nicolè et al. 2012), the intensity of each fragment is transformed into a relative percentage of the highest mass fragment per spectrum. Unsupervised clustering methods are then used to group fragments. SpectConnect (Styczynski et al. 2007) provides feature detection of GC–MS data without requiring use of a reference compound library; instead the user must supply technical replicates of samples. Every spectrum is compared to every other spectrum using the Gemoda (Jensen et al. 2006) algorithm, which finds pairwise similarity of clusters (cliques) using the weighted dot product. The most representative spectra for each clique are chosen, allowing identification of features preserved across samples.

### NMR data processing

There has been far less development of open source software for the analysis of NMR data than for MS. This may be in part due to the majority of NMR spectrometers being supplied by only a few manufacturers. The TopSpin (Bruker BioSpin, Rheinstetten, Germany) software, which comes bundled with Bruker instruments, is the most widely used software tool for NMR metabolomics data preprocessing (Weber et al. 2016). Much of the software that is freely available for use is written in MATLAB (e.g. Dolphin (Gómez et al. 2014), FOCUS (Alonso et al. 2014) and MatNMR (van Beek 2007)), restricting their use to those with access to this costly commercial software. However, compiled versions can be free to use, without requiring a MATLAB license. Gradually MATLAB based tools are being ported onto freely available platforms. Icoshift (Tomasi et al. 2011), a versatile tool for the rapid alignment of 1D NMR spectra now has a Python implementation (mfitzp/icoshift).

The only open software whose use was reported in the Metabolomics Society survey for NMR preprocessing was rNMR (Lewis et al. 2009), which uses a regions of interest (ROIs) based approach for the analysis of 1D and 2D NMR spectra. ROIs can be visually inspected to help aid accurate quantification. The peak lists produced can be directly exported to the Madison Metabolomics Consortium Database (Cui et al. 2008) or uploaded to the Biological Magnetic Resonance Data Bank (BMRB) (Ulrich et al. 2008) for identification.

## Annotation

Metabolite identification remains the most time consuming stage of metabolomics analysis for many users (Weber et al. 2016). It is especially difficult to identify LC–MS features. Only limited structural information can be obtained from mass spectrometry, so it is challenging to identify unknown features. This has been partially solved for GC-MS analysis where extensive commercial libraries (NIST 2014 Reference Database) can be used for identification. Because of this and due to the different order of the GC–MS analysis workflow, there are no tools that specifically designed for GC–MS metabolite identification. Therefore, in this review tools for annotation will simply be split into the MS and NMR categories, depending on which kind of data they are designed for.

Under the existing Metabolomics Standards Initiative (MSI) metabolite identification criteria, for a metabolite to be identified (Level 1), it must be compared to an authentic chemical standard analysed in the same laboratory, using the same analytical techniques as the experimental data (Salek et al. 2013). Thus whilst many metabolomics software purport to offer metabolite identification, they can only provide putative annotation (Level 2). Levels 3 and 4 are putatively characterised compound classes, and unknown compounds respectively.

Alternative criteria proposed by Schymanski et al. (2014) splits MS metabolite identification into five confidence levels. Whilst Level 1 remains unchanged compared to the original MSI criteria, levels 2–5 are different. Probable structure (Level 2) annotation requires either a library spectrum match (2a) or diagnostic evidence (2b). Tentative candidate(s) (Level 3) are for when there is evidence for more than one candidate structure, with inadequate information to narrow identification down to a single structure. Annotation of unequivocal molecular formula (Level 4) requires the use of spectral information for unambiguous assignment.

As many metabolites are not commercially available, they cannot be identified to Level 1. The highest level of identification that can be achieved for these metabolites is Level 2, which is also topmost identification that can be attained using software for identification. Software that provides annotation for Levels 3 and 4 is also available. Software for MS identification will thus be classified by the level of identification provided by Schymanski et al. criteria: Level 4: unequivocal molecular formula, Level 3: tentative candidates and Level 2a: library spectrum match. This criterion was chosen over the original MSI criteria as it provides clearer classification of metabolite annotation assignment confidence.

For NMR identification, the MSI guidelines have also been criticised (Everett 2015). Features can be identified with high confidence using database matching to authentic reference compounds (Dona et al. 2016; Everett 2015), not requiring spectra from an authentic reference standard to be analysed using the same NMR spectrometer. As there are far less tools for NMR metabolite identification than for MS, there will be no further classification subdivision and all software in this category will be included in NMR metabolite identification and quantification.

All of the annotation software included in the text are also listed in Table 2. Further information about all software can be found in Supplementary Table 1, along with 27 additional tools not included in body of the review.

**Table 2:** Software tools commonly used for metabolite annotation

Tool	Annotation level	Software type	Website	References
CAMERA	Level 4	R Package	http://bioconductor.org/packages/release/bioc/html/CAMERA.html	Kuhl et al. (2012)
MZedDB	Level 4	Web App	http://maltese.dbs.aber.ac.uk:8888/hrmet/index.html	Draper et al. (2009)
Rdisop	Level 4	R Package	http://bioconductor.org/packages/release/bioc/html/Rdisop.html	–
SIRIUS	Level 4	CLI, GUI	https://bio.informatik.uni-jena.de/software/sirius	Kim et al. (2016)
MI-PACK	Level 3	CLI	http://www.biosciences-labs.bham.ac.uk/viant/mipack	Weber and Viant (2010)
PUTMEDID-LCMS	Level 3	CLI	http://www.mcisb.org/resources/putmedid.html	Brown et al. (2011)
ProbMetab	Level 3	R Package	http://labpib.fmrp.usp.br/methods/probmetab	Silva et al. (2014)
MetAssign–mzMatch	Level 3	R Package	http://mzmatch.sourceforge.net/index.php	Daly et al. (2014)
MetFrag	Level 2a	Web App	http://c-ruttkies.github.io/MetFrag	Ruttkies et al. (2016)
CFM-ID	Level 2a	CLI, Web App	https://sourceforge.net/projects/cfm-id/	Allen et al. (2014)
FingerID	Level 2a	Web App	https://github.com/icdishb/fingerid	Heinonen et al. (2012)
MAGMa	Level 2a	Web App	http://www.emetabolomics.org/magma	Ridder et al. (2013)
MyCompoundID	Level 2a	Web App	http://mycompoundid.org/mycompoundid_IsoMS	Li et al. (2013)
BATMAN	NMR	R Package	http://batman.r-forge.r-project.org	Hao et al. (2012)
Bayesil	NMR	Web App	http://bayesil.ca	Ravanbakhsh et al. (2015)
MetaboMiner	NMR	CLI	http://wishart.biology.ualberta.ca/metabominer	Xia et al. (2008)
SpinAssign	NMR	Web App	http://prime.psc.riken.jp/?action=nmr_search	Chikayama et al. (2010)
COLMAR	NMR	Web App	http://spin.ccic.ohio-state.edu/index.php/colmar	Zhang et al. (2009)

*CLI* command line interface, *GUI* graphical user interface

### Mass spectrometry

#### Level 4: unequivocal molecular formula

A number of different ionisation products must be identified for the annotation of features in LC–MS data: adducts, isotopes, neutral losses and fragments. Adducts or pseudomolecular ions are the most commonly observed ions in mass spectrometry, due to reactions of metabolites with solvents and metal ions (Keller et al. 2008). It is also important to consider that one or more natural isotopes may be present in every metabolite (Draper et al. 2009). Despite electrospray ionization (ESI) being commonly considered a soft ionisation technique, some metabolites will fragment with neutral losses. This fragmentation has been utilised for MS/MS. However, for MS1 mode data it is important to consider the non-specific fragmentation that occurs.

When there is insufficient evidence to assign a structure to a feature, but adequate information to unambiguously assign a molecular formula, assignments are classified as Level 4 under Schymanski et al.’s (2014) criteria. Molecular formula annotation with adduct, isotope and fragment information is appropriate for low quality MS/MS data and MS data lacking retention time information.

CAMERA is the mostly widely tool used to annotate ionisation products and is in top 5% most downloaded packages in Bioconductor. It can interface directly with XCMS to annotate adducts and common neutral losses. The MZedDB (Draper et al. 2009) database can also be accessed directly from R, allowing for automatic annotation of potential adducts and molecular formulas.

Empirical (or sum) formula annotation provides the relative proportions of the elements in a molecule. Rdisop (Bioconductor—Rdisop) determines a ranked list of potential sum formula of features from high resolution MS data using their exact mass and isotopic patterns. SIRIUS (sum formula identification by ranking isotope patterns using mass spectrometry) (Bocker et al. 2009) resolves the formula of a compound from its fragmented features using PubChem (Kim et al. 2016).

#### Level 3: tentative candidates

Assignment of tentative metabolite candidates does not necessarily require MS/MS data to be available. It can be performed either by manually searching online metabolite databases (HMDB (Wishart et al. 2013), METLIN (Smith et al. 2005), KEGG (Kanehisa et al. 2004, etc.) or automatically using dedicated software tools including: MI-PACK (Weber and Viant 2010) and PUTMEDID-LCMS (Brown et al. 2011). Transformation mapping is used by metabolite identification package (MI-PACK) (Weber and Viant 2010) to putatively annotate metabolites by their interconnectivity in the KEGG database (Kanehisa et al. 2004).

PUTMEDID-LCMS (Brown et al. 2011) is a Taverna based tool, which provides modules that form a workflow for putative metabolite annotation. Correlation analysis is first performed, followed by the annotation of isotopes, adducts, dimers, etc. The Manchester Metabolomics Database built from HMDB (Wishart et al. 2013), KEGG (Kanehisa et al. 2004), LMSD (Sud et al. 2007), BioCyc (Caspi et al. 2016) and DrugBank (Wishart et al. 2006) is then used for putative annotation.

Implemented in R, both MetAssign (Daly et al. 2014) and ProbMetab (Silva et al. 2014) use Bayesian approaches to putatively annotate peaks. MetAssign is a probabilistic putative metabolite identification algorithm, implemented in mzMatch (Scheltema et al. 2011) that uses Bayesian clustering to assign posterior probabilities to the likelihood of the annotation. Features originating from the same metabolite are clustered and annotated as adducts, fragments and isotopes. ProbMetab calculates the likelihood of the assignment of each compound to the target feature using biochemical information, mass accuracy and isotopic carbon pattern if available. The model then uses Gibbs sampling to calculate the posterior probabilities. Metabolites are then directly mapped to pathways, which can optionally be visualised in Cytoscape (Shannon et al. 2003).

#### Level 2a: library spectrum match

A number of online databases, for ESI-MS/MS, MS^n^ and GC–MS, contain spectra acquired using authenticated chemical standards that can be used for performing library spectrum matches. For an extensive review of mass spectral and fragmentation trees see Vaniya and Fiehn (2015). The freely accessible mzCloud (mzCloud—advanced mass spectral database), METLIN (Smith et al. 2005) and MassBank (Horai et al. 2010) databases all contain authenticated MS/MS spectra. Unlike the other spectral databases MassBank allows for the automatic upload of user-generated data to the database, using either the Mass++ (C++) or RMassBank (Stravs et al. 2013) (R) software.

Both ESI-MS/MS and GC–MS spectra acquired using authenticated chemical standards are present in the HMDB (Wishart et al. 2013). Whilst the commercially available NIST 2014 Reference Database historically contained only GC–MS spectra, it now also contains ESI-MS/MS spectra.

A number of software also perform automatic database matching, allowing the user to search multiple MS/MS database simultaneously. Competitive fragment modeling for metabolite identification (CFM-ID) (Allen et al. 2014) annotates ESI-MS/MS. Single energy - competitive fragment modeling (SE-CFM) is used to predict MS/MS spectra at three collision energies: 10 V, 20 V and 40 V. MS/MS spectra can be searched against the HMDB (Wishart et al. 2013) or KEGG databases (Kanehisa et al. 2004) for metabolite identification. FingerID (Heinonen et al. 2012) uses kernel methods to predict a large set of molecular properties for MS/MS matching, searching the PubChem (Kim et al. 2016), MassBank (Horai et al. 2010) and METLIN (Smith et al. 2005) databases. MAGMa (Ridder et al. 2013) generates hierarchical trees in silico for automatic annotation of LC-MS^n^ data, using candidates from PubChem (Kim et al. 2016) and HMDB (Wishart et al. 2013). MetFrag (Ruttkies et al. 2016), another in silico fragmentation tool, has recently been updated to allow users to search a wider selection of databases to identify candidate molecules to generate topological fragments from. Users can also select filtering criteria by inclusion or exclusion of substructures and elements.

The MyCompoundID.org (Li et al. 2013) database encompasses 8021 endogenous human metabolites from HMDB (Wishart et al. 2013) and 375,809 predicted metabolites from the evidence-based metabolome library. It includes an automated MS/MS search program (Huan et al. 2015) that searches a spectral database created using in silico fragmentation prediction, as well as an MS search program. Batch searches can be performed using a CSV of a peak list generated from LC–MS/MS spectral analysis. There are also a number of tools for the identification of specific chemical groups including DnsID (Huan et al. 2015) for dansylate labelled metabolites, PEP (Tang et al. 2014) search for di/tripeptides and IsoMS for isotopic labelling studies.

### NMR metabolite identification and quantification

Compared to the pure reference standard, the majority of chemical shifts of metabolites are within 0.03 ppm for ^1^H NMR and 0.5 ppm for ^13^C NMR (Dona et al. 2016). Due to this low deviation, it has been suggested that for a metabolite to be considered ‘identified’, matching to an authentic compound in a database would be sufficient, provided specific guidelines are followed (Everett 2015). Databases that contain NMR spectra from authentic chemical standards of metabolites include Human Metabolome Database (HMDB) (Wishart et al. 2013), BMRB (Ulrich et al. 2008) and Birmingham Metabolite Library (BML-NMR) (Ludwig et al. 2012). However, despite the consistency of chemical shifts, it remains challenging to identify metabolites that are present at only low levels or which have overlapping signals between multiple metabolites.

NMR is inherently a far more quantitative technique than MS (Emwas 2015). The signal intensity of a feature is directly proportional to the molar concentration of the molecule (Bharti and Roy 2012; Smolinska et al. 2012). However, NMR has limitations in resolution due to the overlaps of signals. This is especially a problem in biofluids, as they are complex mixtures of many compounds and it can be challenging to decipher the molecular concentration for each metabolite (Ellinger et al. 2013). Frequently, the “landmark peak” method is used to determine molecular concentration, although this is not suitable for all ^1^H NMR features, as not all metabolites will have landmark peaks in 1D spectra (Ellinger et al. 2013). Instead spectral libraries, in conjunction with mathematical modelling, are used.

As with preprocessing the majority of researchers use commercial software for NMR metabolite identification and quantification, with Chenomx NMR Suite (Chenomx, Edmonton, Canada) and AMIX (Bruker BioSpin, Rheinstetten, Germany) being the most popular (Weber et al. 2016). Unfortunately many innovations in metabolite identification in NMR data, such as AutoFit (Mercier et al. 2011), are available only with commercial software.

Many of the freely available tools for NMR metabolomics provide both metabolite identification and quantification, with identification and quantification often being performed simultaneously. The BATMAN (Hao et al. 2012) and Bayesil (Ravanbakhsh et al. 2015) software were both reported to be used in the Metabolomics Society survey (Weber et al. 2016). BATMAN (Bayesian automated metabolite analyser for NMR spectra) (Hao et al. 2012) provides a Bayesian model for the deconvolution of ^1^H NMR spectra and a Monte Carlo Markov Chain algorithm to automate metabolite quantification. Metabolites can automatically be identified using a list with user-defined chemical shifts and relative intensity signals for quantification. Bayesil (Ravanbakhsh et al. 2015) is designed to supply automatic spectral processing and identification of serum, plasma and cerebrospinal fluid 1D ^1^H NMR spectra. A reference compound with known concentration is then used for absolute quantification. However, samples must be prepared and spectra must be acquired in a specific way, limiting the use of this software.

Alternative tools include MetaboMiner (Xia et al. 2008), SpinAssign (Chikayama et al. 2010) and COLMAR (Zhang et al. 2009). MetaboMiner performs semi-automated metabolite quantification of 2D TOCSY (TOtal Correlated SpectroscopY) and HSQC (Heteronuclear Single Quantum Coherence) spectra. SpinAssign contains a database of >1700 ^13^C-HSQC peaks, corresponding to 270 metabolites that can be queried for ^1^H and ^13^C chemical shifts, with the percentage match for each putative assignment being calculated. The overlap between the peak of the interest and the reference peak is calculated as the uniqueness score. Complex mixture analysis by NMR (COLMAR) (Zhang et al. 2009) provides three web-servers for the analysis of covariance-NMR (2D) spectra of complex mixtures, which calculate NMR covariance spectra from the raw input, decompose 2D covariance TOCSY spectra into reduced sets of non-redundant 1D cross sections and match traces to the spectral databases, containing spectra from BMRB (Ulrich et al. 2008) and HMDB (Wishart et al. 2013), for metabolite identification.

## Post-processing

Prior to many kinds of statistical analysis metabolomics data must be further wrangled, using post-processing methods, which are alternatively called data pretreatment. These methods encompass data filtering, imputation, normalisation, centering, scaling and transformation. Data can be filtered by applying thresholds to parameters such as signal-to-noise ratio or the minimum percentage of samples a feature must be detected in (consensus features) to remove features which are not found in a minimum number of samples (Alonso et al. 2015). Up to 40% of metabolomics data can be comprised of missing values (Armitage et al. 2015), with a number of causes (Gromski et al. 2014). Imputation is used to ‘fill in’ missing values. Differences in metabolite concentration between samples can be caused by variations in total sample amount and not actual biological variation. It is therefore important to normalise data to minimise the effect of this variation (Wu and Li 2016). Scaling and transformation can change the emphasis to different aspects of the data to enable deciphering of biological information (Gromski et al. 2014; van den Berg et al. 2006).

There are many different techniques for imputation (Gromski et al. 2014; Shah et al. 2015), normalisation (Wu and Li 2016) and scaling (Gromski et al. 2014; van den Berg et al. 2006) and it can be difficult to ascertain the optimal method. Reviews of all of these methods have found there is no ‘ideal’ method that is appropriate for all data, with effects being context dependent (Craig et al. 2006; Di Guida et al. 2016; Gromski et al. 2014; van den Berg et al. 2006; Wu and Li 2016). It is therefore recommended that users try multiple methods to find those that adapt best to their data properties.

The bulk of tools for metabolomics post-processing are available as R packages. This means that both post-processing and the subsequent stage of data processing—statistical analysis, can be performed in the same environment. Some tools combine both of post-processing and statistical analysis, including the metabolomics (De Livera et al. 2012) and muma (Gaude et al. 2013) packages. A list of tools for post-processing can be found in Table 3.

**Table 3:** Software tools for the post-processing of metabolomics data

Tool	Instrument data type	Software type	Website	References
batchCorr	LC–MS	R Package	https://gitlab.com/CarlBrunius/batchCorr	Brunius et al. (2016)
crmn	LC–MS, GC–MS	R Package	https://cran.r-project.org/web/packages/crmn/	Redestig et al. (2009)
EigenMS	LC–MS	CLI	https://sourceforge.net/projects/eigenms	Karpievitch et al. (2014)
KMDA	MS	R Package	https://cran.r-project.org/web/packages/KMDA/	Zhan et al. (2015)
metabolomics	MS, NMR	R Package	https://cran.r-project.org/web/packages/metabolomics/	De Livera et al. (2012)
metabomxtr	LC–MS, GC–MS	R Package	https://www.bioconductor.org/packages/release/bioc/html/metabomxtr.html	Nodzenski et al. (2014)
Metabnorm	NMR	R Script	https://sourceforge.net/projects/metabnorm	Jauhiainen et al. (2014)
MetabR	LC–MS	R Script	http://metabr.r-forge.r-project.org/	Ernest et al. (2012)
MetNorm	LC–MS, GC–MS, NMR	R Package	https://cran.r-project.org/web/packages/MetNorm/	Livera et al. (2015)
MSPrep	LC–MS	R Package	https://sourceforge.net/projects/msprep/	Hughes et al. (2014)
muma	MS, NMR	R Package	https://cran.r-project.org/web/packages/muma/	Gaude et al. (2013)

*CLI* command line interface

## Statistical analysis

After post-processing, data from both MS and NMR studies will be in the form of a matrix of signal intensities. As data from both types of experiment are in the same format, data matrices, the most commonly used techniques are appropriate for both data types. The unsupervised method principal components analysis (PCA) is generally used as an initial exploratory technique. Other supervised methods: partial least squares (PLS) regression (or projection to latent structures), partial least squares-discriminant analysis (PLS-DA) and orthogonal partial least squares (OPLS) are also used. However these techniques have been criticized as they can lead to overfitting (Szymańska et al. 2012; Westerhuis et al. 2008), although validation techniques can be used to evaluate this. More recently other methods are being more widely used as alternatives to PLS-DA (Gromski et al. 2015): principal component-discriminant function analysis (PC-DFA), support vector machines and random forests.

Univariate analyses are also applied, with ANOVA (analysis of variance) and *t*-tests, along with their non-parametric equivalents, being the most widely used (Weber et al. 2016). As these statistical methods are used in many fields, they can be found implemented in many general statistical analysis software applications that are not specifically designed for metabolomics analysis. The R programming language and environment is designed to provide statistical computing and graphics and the majority of statistical analysis methods are implemented in R packages.

An additional statistical technique that is exclusive to NMR is statistical correlation spectroscopy (STOCSY) (Cloarec et al. 2005), which is designed specifically to identify biomarkers from NMR data. STOCSY takes advantage of the multicollinearity of the intensity variables in a set of 1D NMR spectra to generate a pseudo-two-dimensional NMR spectrum that displays the correlation among the intensities of the various peaks across the whole sample. It is particularly good for the identification of metabolites in complex mixtures, such as urine.

Examples of software for metabolomics statistical analysis can be found in Table 4.

**Table 4:** Software tools for the statistical analysis of metabolomics data. CLI - command line interface

Tool	Instrument data type	Software type	Website	References
Ionwinze	LC–MS	R Package	https://sourceforge.net/projects/ionwinze	Kokubun and D’Costa (2013)
MetabolAnalyze	MS, NMR	R Package	https://cran.r-project.org/web/packages/MetabolAnalyze	Nyamundanda et al. (2010)
metabolomics	MS, NMR	R Package	https://cran.r-project.org/web/packages/metabolomics/	De Livera et al. (2012)
MetaboLyzer	MS, NMR	CLI	https://sites.google.com/a/georgetown.edu/fornace-lab-informatics/home/metabolyzer	Mak et al. (2014)
mQTL.NMR	NMR	R Package	https://www.bioconductor.org/packages/release/bioc/html/mQTL.NMR.html	Hedjazi et al. (2015)
muma	MS, NMR	R Package	https://cran.r-project.org/web/packages/muma/	Gaude et al. (2013)
ropls	MS, NMR	R Package	https://www.bioconductor.org/packages/release/bioc/html/ropls.html	Thevenot et al. (2015)

## Workflows

Unlike the previously mentioned software, workflows provide multiple interconnected tools, encompassing all stages of analysis: preprocessing, annotation and statistical analysis. These software are designed for ease-of-use, allowing users to perform the entirety of their analysis using a single tool, rather than having to use separate tools for each stage of the analysis. They also increase data processing and analysis reproducibility. The majority of workflows are provided as web-apps and are primarily designed for the analysis of LC–MS data. The scope of workflows varies a lot, with some including a lot of in-house software and others being workflow management systems, combining existing tools into workflows. Extra information about each software included in this section can be found in Table 5. An extra seven workflows are also included in Supplementary Table 1.

**Table 5:** Workflows for the analysis of metabolomics data

Tool	Instrument data type	Software type	Website	Reference
Workflow4metabolomics	LC–MS, GC–MS	Galaxy	http://workflow4metabolomics.org	Giacomoni et al. (2014)
Galaxy-M	LC–MS	Galaxy	https://github.com/Viant-Metabolomics/Galaxy-M	Davidson et al. (2016)
XCMS Online	LC–MS, GC–MS	Web App	https://xcmsonline.scripps.edu/landing_page.php?pgcontent=mainPage	Tautenhahn et al. (2012)
MetaboAnalyst 3.0	LC–MS	Web App	http://www.metaboanalyst.ca	Xia et al. (2015)
MAVEN	LC–MS	GUI	http://genomics-pubs.princeton.edu/mzroll/index.php	Melamud et al. (2010)
MAIT	LC–MS	R Package	https://www.bioconductor.org/packages/release/bioc/html/MAIT.html	Fernández-Albert et al. (2014)
MZmine 2	LC–MS	GUI	http://mzmine.github.io/	Pluskal et al. (2010)

*GUI* graphical user interface

Galaxy (Afgan et al. 2016) provides a biological workflow platform, allowing for integration of multiple software tools into complete analytical workflows. Although it was originally created for genomics analysis, it is being increasingly used as a general bioinformatics workflow management system. Workflow4metabolomics (Giacomoni et al. 2014) and Galaxy-M (Davidson et al. 2016) are Galaxy-based workflows for the analysis of metabolomics data. Workflow4metabolomics (Giacomoni et al. 2014) encompasses analysis workflows for LC–MS, GC–MS and NMR data, although its LC–MS workflow is the most comprehensive, providing preprocessing, statistical analysis and metabolite annotation. Implementations of the XCMS (Smith et al. 2006), CAMERA (Kuhl et al. 2012) and ropls (Thévenot et al. 2015) packages are included for these analyses. A complete workflow is not available for NMR analysis, however Bruker bucketing and integration, normalisation and statistical analysis are provided. Galaxy-M (Davidson et al. 2016) is designed for the analysis of LC–MS and DIMS metabolomics data, providing preprocessing, statistical analysis and annotation. Like Workflow4metabolomics, Galaxy-M includes installations of XCMS and CAMERA, with MI-PACK (Weber and Viant 2010) additionally available. Also included are a number of imputation, normalisation and filtering methods.

XCMS Online (Tautenhahn et al. 2012) is an online implementation of XCMS (Smith et al. 2006) that includes additional features to incorporate the entire LC–MS data analysis workflow. It differs from XCMS by providing convenient predefined parameters sets for different instrument setups, PCA and univariate statistical analysis and a direct link to the METLIN (Smith et al. 2005) database for putative metabolite annotation. Pathway analysis and data integration with proteomics and transcriptomics data are also supported.

MetaboAnalyst 3.0 (Xia et al. 2015) provides a suite of tools for metabolomics analysis of both MS and NMR data, mainly focused on statistical, enrichment and pathway analysis. It contains eight independent analysis modules composed of three main categories: exploratory statistical analysis, functional analysis and advanced methods for translational studies. Only basic support is provided for the processing of raw data, using the XCMS algorithms for peak picking, grouping and retention time alignment, with only the most commonly used parameters supported.

Metabolomics Analysis and Visualisation ENgine (MAVEN) (Melamud et al. 2010) provides preprocessing, putative metabolite assignment and identification of significant differences between datasets. Peaks are picked, smoothed and grouped, followed by retention time alignment. Peak quality scores are reported to enable to user to identify high quality peaks. Metabolite Automatic Identification Toolkit (MAIT) (Fernández-Albert et al. 2014) provides a wrapper of XCMS (Smith et al. 2006) and CAMERA (Kuhl et al. 2012) for user-friendly LC–MS data analysis. Additionally standard statistical analysis - *t*-tests, ANOVA, PCA and PLS and metabolite annotation via the 2009/07 version of HMDB (Wishart et al. 2013) are also supplied.

MZmine 2 (Pluskal et al. 2010) is the second most used software for LC-MS data preprocessing (Weber et al. 2016), but it also encompasses an entire analysis workflow. Since its initial release, it now includes the GridMass (Treviño et al. 2015) algorithm for feature detection of high resolution liquid chromatography - mass spectrometry (HRLC-MS) data. As of 2017 there are a total of 4 peak detection approaches included in the toolkit. The RANSAC (random sample consensus) or Join aligner algorithms are used for peak alignment (Pluskal et al. 2010). Post-processing, metabolite identification and statistical analysis are additional functions that are also provided.

## Other tools

Some software cannot be easily classified into the previously mentioned categories as they provide other functionalities. These tools relate to improving experimental design and optimising parameters, both instrumental and software based. There are tools designed to optimise feature detection of LC–MS(/MS) data, requiring the user to perform experiments in a specified way (Mahieu et al. 2014; Neumann et al. 2012). In addition there are tools for estimating the required sample size for achieving sufficient power (Nyamundanda et al. 2013). Tools classified as Other tools do not have a standardised place in the analysis pipeline and where they fit into the pipeline depends on their functionality. Fourteen tools are classified as Other Tools and are included in the supplementary material.

## Future prospects

This review presents the most widely used tools for metabolomics analysis, categorised based on their main functionality. As it is beyond the scope of this review, there has been no direct comparison of tools, resulting in a ranked list of the ‘best’ tools to perform a specific data analysis task e.g. peak picking. In future it would be beneficial for systematic reviews, comparing large numbers of freely available tools designed for specific tasks in metabolomics data analysis, using benchmarked datasets containing only known metabolites. Whilst there have been some reviews comparing the accuracy of peak picking of a number of software for LC–MS and GC–MS, these reviews have mostly focused on commercial software (Rafiei and Sleno 2015), have not optimised software parameters (Coble and Fraga 2014) or have not used MS/MS data (Lange et al. 2008). This is especially important for NMR-based metabolomics where no such review has yet been conducted.

OMICtools (Henry et al. 2014) is a manually curated metadatabase of tools for the analysis of omics data, containing both commercial and open source software. Whilst it provides a lot of useful information about software beyond its functionality, including computer skills required, licensing, programming languages and interfaces, it does not containing other information that a user will require when deciding which tools to use, such as input formats. It is also missing a lot of the most recently released software.

Ms-utils (ms-utils.org—Software List) provides a list of tools for mass spectrometry data analysis, but it is mainly focused on proteomics. The Fiehn lab website (Metabolomics—Fiehn Lab) and the metabolomics society webpage (Metabolomics Society: Metabolomics Software and Servers) also contain lists of metabolomics software. However, again these are not comprehensive lists, which are not updated to include the mostly recently released tools. Supplementary Table 1 of this review and https://github.com/RASpicer/MetabolomicsTools includes a comprehensive list of tools, along with details of their functionality, operating systems they run on and installation requirements. However, this does not include all tools for metabolomics analysis and more tools are constantly released.

In the CASMI (Critical Assessment of Small Molecule Identification) (Schymanski and Neumann 2016) competition, teams compete in a series of challenges to identify as many small molecules as possible. The Best Automatic Structural Identification categories directly compare tools small molecule identification. In 2016 the categories were split into in silico fragmentation only and tools used along with additional information e.g. retention time. MS-FINDER (Tsugawa et al. 2016) and CFM-ID (Allen et al. 2014) were used by the teams who came 1st and 2nd respectively in the Full Information category and IOKR (Brouard et al. 2016) and fingerID (Heinonen et al. 2012) were used by the two top teams in the in silico fragmentation category.

Ideally a novel database of software for metabolomics data analysis would be created. This should allow users to manually add their newly released tools to inform the community about them. It should include specifically which tools are maintained i.e. automatically acquiring when the last update time, as well as important information such as input formats and skill level required.

A new database could also help to address the lack of compatibility between tools. Currently in metabolomics there is a major problem of interoperability between tools for the different steps of data analysis: the output of one tool not being an acceptable input format for other tools for the subsequent stages of analysis. There can also be incompatibility between dependencies and required software versions. By directly reporting compatible tools, the database could help users with this issue.

Tool harmonisation is also being improved by efforts to containerise tools, such as those by the PhenoMeNal consortium (PhenoMeNal) and the BioContainers initiative (Leprevost et al. 2017). Creating containers for tools (or Dockerising (Docker)) isolates them and their dependencies in terms of installation, which removes incompatibility between tools caused by dependencies and varying versions. Because the usual practice is to deposit built container images with explicit versions into publicly available online container registries (such as Docker Hub or quay.io), older versions of a container used in past analysis can always be retrieved to reproduce it, with the same analysis tools with exactly the same versions used as the original analysis (provided this was done through a container). This improves both the accessibility of tools and reproducibility of data analysis.

## Electronic supplementary material

Below is the link to the electronic supplementary material.

Supplementary material 1 (XLSX 174 KB)

## References

[CR1] Afgan E, Baker D, van den Beek M, Blankenberg D, Bouvier D, Čech M (2016). The Galaxy platform for accessible, reproducible and collaborative biomedical analyses: 2016 update. Nucleic Acids Research.

[CR2] Allen F, Pon A, Wilson M, Greiner R, Wishart D (2014). CFM-ID: A web server for annotation, spectrum prediction and metabolite identification from tandem mass spectra. Nucleic Acids Research.

[CR3] Alonso A, Marsal S, Julià A (2015). Analytical methods in untargeted metabolomics: State of the art in 2015. Frontiers in Bioengineering and Biotechnology.

[CR4] Alonso A, Rodríguez Ma, Vinaixa M, Tortosa R, Correig X, Julià A, Marsal S (2014). Focus: A robust workflow for one-dimensional NMR spectral analysis. Analytical Chemistry.

[CR5] Armitage EG, Godzien J, Alonso-Herranz V, López-Gonzálvez Á, Barbas C (2015). Missing value imputation strategies for metabolomics data. Electrophoresis.

[CR6] Bertsch A, Gröpl C, Reinert K, Kohlbacher O, Hamacher M, Eisenacher M, Stephan C (2010). OpenMS and TOPP: Open source software for LC-MS data analysis. Data mining in proteomics.

[CR7] Bioconductor - Rdisop. (2016). Accessed August 18, 2016 from http://bioconductor.org/packages/release/bioc/html/Rdisop.html.

[CR8] Bharti SK, Roy R (2012). Quantitative 1H NMR spectroscopy. Trends in Analytical Chemistry.

[CR9] Bocker S, Letzel MC, Liptak Z, Pervukhin A (2009). SIRIUS: decomposing isotope patterns for metabolite identification. Bioinformatics.

[CR10] Booth SC, Weljie AM, Turner RJ (2013). Computational tools for the secondary analysis of metabolomics experiments. Computational and Structural Biotechnology Journal.

[CR12] Broeckling CD, Reddy IR, Duran AL, Zhao X, Sumner LW, Division PB (2006). MET-IDEA: Data extraction tool for mass spectrometry-based metabolomics. Analytical Chemistry.

[CR13] Brouard C, Shen H, Dührkop K, d’Alché-Buc F, Böcker S, Rousu J (2016). Fast metabolite identification with input output kernel regression. Bioinformatics.

[CR14] Brown M, Wedge DC, Goodacre R, Kell DB, Baker PN, Kenny LC (2011). Automated workflows for accurate mass-based putative metabolite identification in LC/MS-derived metabolomic datasets. Bioinformatics.

[CR15] Brunius C, Shi L, Landberg R (2016). Large-scale untargeted LC-MS metabolomics data correction using between-batch feature alignment and cluster-based within-batch signal intensity drift correction. Metabolomics.

[CR16] Caspi R, Billington R, Ferrer L, Foerster H, Fulcher CA, Keseler IM (2016). The MetaCyc database of metabolic pathways and enzymes and the BioCyc collection of pathway/genome databases. Nucleic Acids Research.

[CR17] Chambers MC, Maclean B, Burke R, Amodei D, Ruderman DL, Neumann S (2012). A cross-platform toolkit for mass spectrometry and proteomics. Nature Biotechnology.

[CR18] Chen, Y. T., Zhang, J., Zhang, X., & Kim, S. (2011). Statistical Analysis of Gas Chromatography Retention Index Database. In *2011 5th International Conference on Bioinformatics and Biomedical Engineering* (pp. 1–4).

[CR19] Chikayama E, Sekiyama Y, Okamoto M, Nakanishi Y, Tsuboi Y, Akiyama K (2010). Statistical indices for simultaneous large-scale metabolite detections for a single NMR spectrum. Analytical Chemistry.

[CR20] Chokkathukalam A, Jankevics A, Creek DJ, Achcar F, Barrett MP, Breitling R (2013). mzMatch–ISO: an R tool for the annotation and relative quantification of isotope-labelled mass spectrometry data. Bioinformatics.

[CR21] Cloarec O, Dumas M-E, Craig A, Barton RH, Trygg J, Hudson J (2005). Statistical total correlation spectroscopy: An exploratory approach for latent biomarker identification from metabolic 1 H NMR data sets. Analytical Chemistry.

[CR22] Coble JB, Fraga CG (2014). Comparative evaluation of preprocessing freeware on chromatography/mass spectrometry data for signature discovery. Journal of chromatography A.

[CR23] Conley, C. J., Smith, R., Torgrip, R. J. O., Taylor, R. M., Tautenhahn, R., & Prince, J. T. (2014). Massifquant: open-source Kalman filter-based XC-MS isotope trace feature detection. *Bioinformatics*, 1–8. 10.1093/bioinformatics/btu359 24872423

[CR24] Craig A, Cloarec O, Holmes E, Nicholson JK, Lindon JC (2006). Scaling and normalization effects in NMR spectroscopic metabonomic data sets. Analytical Chemistry.

[CR25] Creek DJ, Jankevics A, Burgess KEV, Breitling R, Barrett MP (2012). IDEOM: an Excel interface for analysis of LC-MS-based metabolomics data. Bioinformatics.

[CR26] Cui Q, Lewis IA, Hegeman AD, Anderson ME, Li J, Schulte CF (2008). Metabolite identification via the Madison Metabolomics Consortium Database. Nature Biotechnology.

[CR27] Daly R, Rogers S, Wandy J, Jankevics A, Burgess KEV, Breitling R (2014). MetAssign: Probabilistic annotation of metabolites from LC–MS data using a Bayesian clustering approach. Bioinformatics.

[CR50] da Leprevost V, Grüning BA, Alves Aflitos S, Röst HL, Uszkoreit J, Barsnes H (2017). BioContainers: An open-source and community-driven framework for software standardization. Bioinformatics.

[CR28] Davidson RL, Weber RJM, Liu H, Sharma-Oates A, Viant MR (2016). Galaxy-M: A Galaxy workflow for processing and analyzing direct infusion and liquid chromatography mass spectrometry-based metabolomics data. GigaScience.

[CR29] De Livera AM, Dias DA, De Souza D, Rupasinghe T, Pyke J, Tull D (2012). Normalizing and integrating metabolomics data. Analytical Chemistry.

[CR30] Di Guida R, Engel J, Allwood JW, Weber RJM, Jones MR, Sommer U (2016). Non-targeted UHPLC-MS metabolomic data processing methods: A comparative investigation of normalisation, missing value imputation, transformation and scaling. Metabolomics.

[CR31] Docker (2017). Accessed 6 July 2017 from https://www.docker.com/.

[CR32] Domingo-Almenara X, Brezmes J, Vinaixa M, Samino S, Ramirez N, Ramon-Krauel M (2016). eRah: A computational tool integrating spectral deconvolution and alignment with quantification and identification of metabolites in GC/MS-based metabolomics. Analytical Chemistry.

[CR33] Dona AC, Kyriakides M, Scott F, Shephard EA, Varshavi D, Veselkov K, Everett JR (2016). A guide to the identification of metabolites in NMR-based metabonomics/metabolomics experiments. Computational and Structural Biotechnology Journal.

[CR34] Döös K, Kjellsson J, Jönsson B, Soomere T, Quak E (2013). TRACMASS—A Lagrangian trajectory model. Preventive methods for coastal protection.

[CR35] Draper J, Enot DP, Parker D, Beckmann M, Snowdon S, Lin W, Zubair H (2009). Metabolite signal identification in accurate mass metabolomics data with MZedDB, an interactive m/z annotation tool utilising predicted ionisation behaviour “rules.”. BMC Bioinformatics.

[CR36] Du P, Kibbe Wa, Lin SM (2006). Improved peak detection in mass spectrum by incorporating continuous wavelet transform-based pattern matching. Bioinformatics.

[CR37] Earll, M. (2012). Open source software for mass spectrometry and metabolomics. In *Open source software in life science research: Practical solutions to common challenges in the pharmaceutical industry and beyond* (pp. 89–129).

[CR38] Ellinger JJ, Chylla RA, Ulrich EL, Markley JL (2013). Databases and software for NMR-based metabolomics. Current Metabolomics.

[CR39] Emwas A-H M (2015). The strengths and weaknesses of NMR spectroscopy and mass spectrometry with particular focus on metabolomics research. Methods in Molecular Biology.

[CR40] Ernest B, Gooding JR, Campagna SR, Saxton AM, Voy BH (2012). MetabR: An R script for linear model analysis of quantitative metabolomic data. BMC Research Notes.

[CR41] Everett JR (2015). A new paradigm for known metabolite identification in metabonomics/metabolomics: Metabolite identification efficiency. Computational and Structural Biotechnology Journal.

[CR42] Fernández-Albert F, Llorach R, Andrés-Lacueva C, Perera A (2014). An R package to analyse LC/MS metabolomic data: MAIT (Metabolite Automatic Identification Toolkit). Bioinformatics.

[CR43] Fiehn, O. (2002). Metabolomics-the link between genotypes and phenotypes. *Plant Molecular Biology*. http://link.springer.com/article/10.1023/A:1013713905833. 11860207

[CR44] Gaude E, Chignola F, Spiliotopoulos D, Spitaleri A, Ghitti M, Garcia-Manteiga JM (2013). muma, An R package for metabolomics univariate and multivariate statistical analysis. Current Metabolomics.

[CR45] Giacomoni F, Corguillé GL, Monsoor M, Landi M, Pericard P, Pétéra M (2014). Workflow4Metabolomics: A collaborative research infrastructure for computational metabolomics. Bioinformatics.

[CR46] Gómez J, Brezmes J, Mallol R, Rodríguez MA, Vinaixa M, Salek RM (2014). Dolphin: A tool for automatic targeted metabolite profiling using 1D and 2D 1H-NMR data. Analytical and Bioanalytical Chemistry.

[CR47] Gromski PS, Muhamadali H, Ellis DI, Xu Y, Correa E, Turner ML, Goodacre R (2015). A tutorial review: Metabolomics and partial least squares-discriminant analysis—a marriage of convenience or a shotgun wedding. Analytica Chimica Acta.

[CR48] Gromski PS, Xu Y, Hollywood KA, Turner ML, Goodacre R (2014). The influence of scaling metabolomics data on model classification accuracy. Metabolomics.

[CR49] Gromski PS, Xu Y, Kotze HL, Correa E, Ellis DI, Armitage EG (2014). Influence of missing values substitutes on multivariate analysis of metabolomics data. Metabolites.

[CR51] Hao J, Astle W, De iorio M, Ebbels TMD (2012). Batman: An R package for the automated quantification of metabolites from nuclear magnetic resonance spectra using a bayesian model. Bioinformatics.

[CR52] Haug K, Salek RM, Conesa P, Hastings J, de Matos P, Rijnbeek M (2013). MetaboLights–an open-access general-purpose repository for metabolomics studies and associated meta-data. Nucleic Acids Research.

[CR53] Hedjazi L, Gauguier D, Zalloua PA, Nicholson JK, Dumas M-E, Cazier J-B (2015). mQTL.NMR: An integrated suite for genetic mapping of quantitative variations of (1)H NMR-based metabolic profiles. Analytical Chemistry.

[CR54] Heinonen M, Shen H, Zamboni N, Rousu J (2012). Metabolite identification and molecular fingerprint prediction through machine learning. Bioinformatics.

[CR55] Henry VJ, Bandrowski AE, Pepin A-S, Gonzalez BJ, Desfeux A (2014). OMICtools: An informative directory for multi-omic data analysis. Database: The Journal of Biological Databases and Curation.

[CR56] Hiller K, Hangebrauk J, Jäger C, Spura J, Schreiber K, Schomburg D (2009). Metabolite detector: Comprehensive analysis tool for targeted and nontargeted GC/MS based metabolome analysis. Analytical Chemistry.

[CR57] Horai H, Arita M, Kanaya S, Nihei Y, Ikeda T, Suwa K (2010). MassBank: A public repository for sharing mass spectral data for life sciences. Journal of Mass Spectrometry.

[CR58] Huan T, Tang C, Li R, Shi Y, Lin G, Li L (2015). MyCompoundID MS/MS Search: Metabolite identification using a library of predicted fragment-ion-spectra of 383,830 possible human metabolites. Analytical Chemistry.

[CR59] Huan T, Wu Y, Tang C, Lin G, Li L (2015). DnsID in MyCompoundID for rapid identification of dansylated amine- and phenol-containing metabolites in LC–MS-based metabolomics. Analytical Chemistry.

[CR60] Hughes G, Cruickshank-Quinn C, Reisdorph R, Lutz S, Petrache I, Reisdorph N (2014). MSPrep–summarization, normalization and diagnostics for processing of mass spectrometry-based metabolomic data. Bioinformatics.

[CR61] Jauhiainen A, Madhu B, Narita M, Narita M, Griffiths J, Tavaré S (2014). Normalization of metabolomics data with applications to correlation maps. Bioinformatics.

[CR62] Jensen KL, Styczynski MP, Rigoutsos I, Stephanopoulos GN (2006). A generic motif discovery algorithm for sequential data. Bioinformatics.

[CR63] Kanehisa M, Goto S, Kawashima S, Okuno Y, Hattori M (2004). The KEGG resource for deciphering the genome. Nucleic Acids Research.

[CR64] Karpievitch YV, Nikolic SB, Wilson R, Sharman JE, Edwards LM (2014). Metabolomics data normalization with EigenMS. PLoS One.

[CR65] Kell DB, Oliver SG (2016). The metabolome 18 years on: A concept comes of age. Metabolomics.

[CR66] Keller BO, Sui J, Young AB, Whittal RM (2008). Interferences and contaminants encountered in modern mass spectrometry. Analytica Chimica Acta.

[CR67] Kenar E, Franken H, Forcisi S, Wormann K, Haring HU, Lehmann R (2014). Metabolites from liquid chromatography–mass spectrometry data. Molecular & Cellular Proteomics.

[CR68] Kessler N, Neuweger H, Bonte A, Langenkämper G, Niehaus K, Nattkemper TW, Goesmann A (2013). MeltDB 2.0-advances of the metabolomics software system. Bioinformatics.

[CR69] Kim S, Thiessen PA, Bolton EE, Chen J, Fu G, Gindulyte A (2016). PubChem substance and compound databases. Nucleic Acids Research.

[CR70] Kokubun T, D’Costa L (2013). Direct and unbiased information recovery from liquid chromatography-mass spectrometry raw data for phenotype-differentiating metabolites based on screening window coefficient of ion currents. Analytical Chemistry.

[CR71] Kuhl C, Tautenhahn R, Böttcher C, Larson TR, Neumann S (2012). CAMERA: An integrated strategy for compound spectra extraction and annotation of liquid chromatography/mass spectrometry data sets. Analytical Chemistry.

[CR72] Metabolomics - Fiehn Lab. (2016). Accessed September 28, 2016 from http://fiehnlab.ucdavis.edu/staff/kind/Metabolomics.

[CR73] Lange E, Tautenhahn R, Neumann S, Gröpl C (2008). Critical assessment of alignment procedures for LC-MS proteomics and metabolomics measurements. BMC Bioinformatics.

[CR74] Lewis Ia, Schommer SC, Markley JL (2009). rNMR: Open source software for identifying and quantifying metabolites in NMR spectra. Magnetic Resonance in Chemistry.

[CR75] Li L, Li R, Zhou J, Zuniga A, Stanislaus AE, Wu Y (2013). MyCompoundID: Using an evidence-based metabolome library for metabolite identification. Analytical Chemistry.

[CR76] Livera AMD, Sysi-Aho M, Jacob L, Gagnon-Bartsch JA, Castillo S, Simpson JA, Speed TP (2015). Statistical methods for handling unwanted variation in metabolomics data. Analytical Chemistry.

[CR77] Lommen A, Kools HJ (2012). MetAlign 3.0: Performance enhancement by efficient use of advances in computer hardware. Metabolomics.

[CR78] Ludwig C, Easton JM, Lodi A, Tiziani S, Manzoor SE, Southam AD (2012). Birmingham metabolite library: A publicly accessible database of 1-D 1 H and 2-D 1 H J-resolved NMR spectra of authentic metabolite standards (BML-NMR). Metabolomics.

[CR79] Mahieu NG, Huang X, Chen Y-J, Patti GJ (2014). Credentialing features: a platform to benchmark and optimize untargeted metabolomic methods. Analytical Chemistry.

[CR80] Mak TD, Laiakis EC, Goudarzi M, Fornace AJ (2014). MetaboLyzer: A novel statistical workflow for analyzing postprocessed LC-MS metabolomics data. Analytical Chemistry.

[CR81] Martens L, Chambers M, Sturm M, Kessner D, Levander F, Shofstahl J (2011). mzML—a community standard for mass spectrometry data. Molecular & Cellular Proteomics.

[CR82] MATLAB - MathWorks. (2016). Accessed September 14, 2016 from http://www.mathworks.com/products/matlab/.

[CR83] Melamud E, Vastag L, Rabinowitz JD (2010). Metabolomic analysis and visualization engine for LC-MS data. Analytical Chemistry.

[CR84] Mercier P, Lewis MJ, Chang D, Baker D, Wishart DS (2011). Towards automatic metabolomic profiling of high-resolution one-dimensional proton NMR spectra. Journal of Biomolecular NMR.

[CR85] Metabolomics Society: Metabolomics Software and Servers. (2016). http://metabolomicssociety.org/resources/metabolomics-software. Accessed September 5, 2016.

[CR86] Meyer MR, Peters FT, Maurer HH (2010). Automated mass spectral deconvolution and identification system for GC-MS screening for drugs, poisons, and metabolites in urine. Clinical Chemistry.

[CR87] Misra BB, van der Hooft JJJ (2016). Updates in metabolomics tools and resources: 2014–2015. Electrophoresis.

[CR88] Morris GA (2017). NMR data processing. Encyclopedia of Spectroscopy and Spectrometry.

[CR89] mfitzp/icoshift - GitHub. (2017). Accessed January 31, 2017 from https://github.com/mfitzp/icoshift.

[CR90] ms-utils.org - Software List. (2016). Accessed November 23, 2016 from http://www.ms-utils.org/.

[CR91] mzCloud - advanced mass spectral database. (2016). Accessed September 15, 2016 from https://www.mzcloud.org/.

[CR92] Neumann, S., Thum, A., & Böttcher, C. (2012). Nearline acquisition and processing of liquid chromatography-tandem mass spectrometry data. *Metabolomics*, 84–91.

[CR93] Nicolè F, Guitton Y, Courtois EA, Moja S, Legendre L, Hossaert-Mckey M (2012). MSeasy: Unsupervised and untargeted GC-MS data processing. Bioinformatics.

[CR94] Niu W, Knight E, Xia Q, McGarvey BD (2014). Comparative evaluation of eight software programs for alignment of gas chromatography–mass spectrometry chromatograms in metabolomics experiments. Journal of Chromatography A.

[CR95] NIST Standard Reference Database 1 A v14. (2016). Accessed August 20, 2016 from http://www.nist.gov/srd/nist1a.cfm.

[CR96] Nodzenski M, Muehlbauer MJ, Bain JR, Lowe WL, Scholtens DM (2014). Metabomxtr: An R package for mixture-model analysis of non-targeted metabolomics data. Bioinformatics.

[CR97] Nyamundanda G, Brennan L, Gormley IC (2010). Probabilistic principal component analysis for metabolomic data. BMC Bioinformatics.

[CR98] Nyamundanda G, Gormley IC, Fan Y, Gallagher WM, Brennan L (2013). MetSizeR: selecting the optimal sample size for metabolomic studies using an analysis based approach. BMC Bioinformatics.

[CR99] GNU Octave. (2017). Accessed March 28, 2017 from https://www.gnu.org/software/octave/.

[CR100] Pedrioli PGA, Eng JK, Hubley R, Vogelzang M, Deutsch EW, Raught B (2004). A common open representation of mass spectrometry data and its application to proteomics research. Nature Biotechnology.

[CR101] PhenoMeNal. (2017). Accessed June 2, 2017 from http://phenomenal-h2020.eu.

[CR102] Pluskal T, Castillo S, Villar-Briones A, Oresic M (2010). MZmine 2: modular framework for processing, visualizing, and analyzing mass spectrometry-based molecular profile data. BMC Bioinformatics.

[CR103] Prince JT, Marcotte EM (2006). Chromatographic alignment of ESI-LC-MS proteomics data sets by ordered bijective interpolated warping. Analytical Chemistry.

[CR104] Rafiei A, Sleno L (2015). Comparison of peak-picking workflows for untargeted liquid chromatography/high-resolution mass spectrometry metabolomics data analysis. Rapid Communications in Mass Spectrometry.

[CR105] Ravanbakhsh S, Liu P, Bjordahl TC, Mandal R, Grant JR, Wilson M (2015). Accurate, fully-automated NMR spectral profiling for metabolomics. PLoS ONE.

[CR106] Redestig H, Fukushima A, Stenlund H, Moritz T, Arita M, Saito K, Kusano M (2009). Compensation for systematic cross-contribution improves normalization of mass spectrometry based metabolomics data. Analytical Chemistry.

[CR107] Ren S, Hinzman AA, Kang EL, Szczesniak RD, Lu LJ (2015). Computational and statistical analysis of metabolomics data. Metabolomics.

[CR108] Rew R, Davis G (1990). NetCDF: An interface for scientific data access. IEEE Computer Graphics and Applications.

[CR109] Ridder L, van der Hooft JJJ, Verhoeven S, de Vos RCH, Bino RJ, Vervoort J (2013). Automatic chemical structure annotation of an LC–MSn based metabolic profile from green tea. Analytical Chemistry.

[CR110] Rocca-Serra P, Brandizi M, Maguire E, Sklyar N, Taylor C, Begley K (2010). ISA software suite: Supporting standards-compliant experimental annotation and enabling curation at the community level. Bioinformatics.

[CR111] Ruttkies C, Schymanski EL, Wolf S, Hollender J, Neumann S (2016). MetFrag relaunched: Incorporating strategies beyond in silico fragmentation. Journal of Cheminformatics.

[CR112] Salek RM, Steinbeck C, Viant MR, Goodacre R, Dunn WB (2013). The role of reporting standards for metabolite annotation and identification in metabolomic studies. GigaScience.

[CR113] Scheltema, Jankevics, Jansen, R C, Swertz Ma, &amp, Breitling R (2011). PeakML/mzMatch: A file format, Java library, R library, and tool-chain for mass spectrometry data analysis. Analytical Chemistry.

[CR114] Schymanski, E., & Neumann, S. (2016). Critical assessment of small molecule identification. *Metabolites, 3*, 517–538. Accessed September 28, 2016 from http://casmi-contest.org/2016/index.shtml. 10.3390/metabo3030517 PMC3901296 24958137

[CR115] Schymanski EL, Jeon J, Gulde R, Fenner K, Ruff M, Singer HP, Hollender J (2014). Identifying small molecules via high resolution mass spectrometry: communicating confidence. Environmental Science & Technology.

[CR116] Shah JS, Brock GN, Rai SN (2015). Metabolomics data analysis and missing value issues with application to infarcted mouse hearts. BMC bioinformatics.

[CR117] Shannon P, Markiel A, Ozier O, Baliga NS, Wang JT, Ramage D (2003). Cytoscape: A software environment for integrated models of biomolecular interaction networks. Genome Research.

[CR118] Silva RR, Jourdan F, Salvanha DM, Letisse F, Jamin EL, Guidetti-Gonzalez S (2014). ProbMetab: an R package for Bayesian probabilistic annotation of LC–MS-based metabolomics. Bioinformatics.

[CR119] Smith CA, O’Maille G, Want EJ, Qin C, Trauger SA, Brandon TR (2005). METLIN: a metabolite mass spectral database. Therapeutic Drug Monitoring.

[CR120] Smith CA, Want EJ, O’Maille G, Abagyan R, Siuzdak G (2006). XCMS: processing mass spectrometry data for metabolite profiling using nonlinear peak alignment, matching, and identification. ACS Publications.

[CR121] Smolinska A, Blanchet L, Buydens LMC, Wijmenga SS (2012). NMR and pattern recognition methods in metabolomics: From data acquisition to biomarker discovery: A review. Analytica Chimica Acta.

[CR122] Stravs MA, Schymanski, E L, Singer HP, &amp, Hollender J (2013). Automatic recalibration and processing of tandem mass spectra using formula annotation. Journal of Mass Spectrometry.

[CR123] Styczynski MP, Moxley JF, Tong LV, Walther JL, Jensen KL, Stephanopoulos GN (2007). Systematic identification of conserved metabolites in GC/MS data for metabolomics and biomarker discovery. Analytical Chemistry.

[CR124] Sud M, Fahy E, Cotter D, Azam K, Vadivelu I, Burant C (2016). Metabolomics Workbench: An international repository for metabolomics data and metadata, metabolite standards, protocols, tutorials and training, and analysis tools. Nucleic Acids Research.

[CR125] Sud M, Fahy E, Cotter D, Brown A, Dennis EA, Glass CK (2007). LMSD: LIPID MAPS structure database. Nucleic Acids Research.

[CR126] Sugimoto M, Kawakami M, Robert M, Soga T, Tomita M (2012). Bioinformatics tools for mass spectroscopy-based metabolomic data processing and analysis. Current Bioinformatics.

[CR127] Szymańska E, Saccenti E, Smilde AK, Westerhuis JA (2012). Double-check: Validation of diagnostic statistics for PLS-DA models in metabolomics studies. Metabolomics.

[CR128] Tang Y, Li R, Lin G, Li L (2014). PEP search in MyCompoundID: detection and identification of dipeptides and tripeptides using dimethyl labeling and hydrophilic interaction liquid chromatography tandem mass spectrometry. Analytical Chemistry.

[CR129] Tautenhahn R, Böttcher C, Neumann S (2008). Highly sensitive feature detection for high resolution LC/MS. BMC Bioinformatics.

[CR130] Tautenhahn R, Patti GJ, Rinehart D, Siuzdak G (2012). XCMS Online: A web-based platform to process untargeted metabolomic data. Analytical Chemistry.

[CR131] Thévenot EA, Roux A, Xu Y, Ezan E, Junot C (2015). Analysis of the human adult urinary metabolome variations with age, body mass index, and gender by implementing a comprehensive workflow for univariate and OPLS statistical analyses. Journal of Proteome Research.

[CR132] Tomasi G, Savorani F, Engelsen SB (2011). Icoshift: An effective tool for the alignment of chromatographic data. Journal of Chromatography A.

[CR133] Treutler H, Neumann S (2016). Prediction, detection, and validation of isotope clusters in mass spectrometry data. Metabolites.

[CR134] Treviño V, Yañez-Garza I-L, Rodriguez-López CE, Urrea-López R, Garza-Rodriguez M-L, Tamez-Peña JG (2015). GridMass: A fast two-dimensional feature detection method for LC/MS. Journal of Mass Spectrometry.

[CR135] Tsugawa H, Cajka T, Kind T, Ma Y, Higgins B, Ikeda K (2015). MS-DIAL: Data-independent MS/MS deconvolution for comprehensive metabolome analysis. Nature Methods.

[CR136] Tsugawa H, Kind T, Nakabayashi R, Yukihira D, Tanaka W, Cajka T (2016). Hydrogen rearrangement rules: Computational MS/MS fragmentation and structure elucidation using MS-FINDER software. Analytical Chemistry.

[CR137] Turewicz M, Deutsch EW, Hamacher M, Eisenacher M, Stephan C (2010). Spectra, chromatograms, metadata: mzML-the standard data format for mass spectrometer output. Data mining in proteomics.

[CR138] Ulrich EL, Akutsu H, Doreleijers JF, Harano Y, Ioannidis YE, Lin J (2008). BioMagResBank. Nucleic Acids Research.

[CR139] van Beek JD (2007). matNMR: A flexible toolbox for processing, analyzing and visualizing magnetic resonance data in Matlab. Journal of Magnetic Resonance.

[CR140] van den Berg RA, Hoefsloot HCJ, Westerhuis JA, Smilde AK, van der Werf MJ (2006). Centering, scaling, and transformations: Improving the biological information content of metabolomics data. BMC Genomics.

[CR141] Vaniya A, Fiehn O (2015). Using fragmentation trees and mass spectral trees for identifying unknown compounds in metabolomics. Trends in analytical chemistry: TRAC.

[CR142] Vettukattil R (2015). Preprocessing of raw metabonomic data. Methods in Molecular Biology.

[CR143] Want E, Masson P (2011). Processing and analysis of GC/LC-MS-based metabolomics data. Methods in Molecular Biology.

[CR144] Weber RJM, Lawson TN, Salek RM, Ebbels TMD, Glen RC, Goodacre R (2016). Computational tools and workflows in metabolomics: An international survey highlights the opportunity for harmonisation through Galaxy. Metabolomics.

[CR145] Weber RJM, Viant MR (2010). MI-Pack: Increased confidence of metabolite identification in mass spectra by integrating accurate masses and metabolic pathways. Chemometrics and Intelligent Laboratory Systems.

[CR146] Wehrens R, Weingart G, Mattivi F (2014). metaMS: An open-source pipeline for GC–MS-based untargeted metabolomics. Journal of Chromatography B.

[CR147] Westerhuis JA, Hoefsloot HCJ, Smit S, Vis DJ, Smilde AK, van Velzen EJJ (2008). Assessment of PLSDA cross validation. Metabolomics.

[CR148] Winnike JH, Wei X, Knagge KJ, Colman SD, Gregory SG, Zhang X (2015). Comparison of GC-MS and GC × GC-MS in the analysis of human serum samples for biomarker discovery. Journal of Proteome Research.

[CR149] Wishart DS, Jewison T, Guo AC, Wilson M, Knox C, Liu Y (2013). HMDB 3.0-The human metabolome database in 2013. Nucleic Acids Research.

[CR150] Wishart DS, Knox C, Guo AC, Shrivastava S, Hassanali M, Stothard P (2006). DrugBank: A comprehensive resource for in silico drug discovery and exploration. Nucleic Acids Research.

[CR151] Wolfram Mathematica: Modern technical computing. (2016). Accessed September 14, 2016 from https://www.wolfram.com/mathematica/.

[CR152] Wu Y, Li L (2016). Sample normalization methods in quantitative metabolomics. Journal of Chromatography A.

[CR153] Xia J, Bjorndahl TC, Tang P, Wishart DS (2008). MetaboMiner—semi-automated identification of metabolites from 2D NMR spectra of complex biofluids. BMC Bioinformatics.

[CR154] Xia J, Sinelnikov IV, Han B, Wishart DS (2015). MetaboAnalyst 3.0—making metabolomics more meaningful. Nucleic Acids Research.

[CR155] Zhan X, Patterson AD, Ghosh D (2015). Kernel approaches for differential expression analysis of mass spectrometry-based metabolomics data. BMC Bioinformatics.

[CR156] Zhang F, Robinette SL, Bruschweiler-Li L, Brüschweiler R (2009). Web server suite for complex mixture analysis by covariance NMR. Magnetic Resonance in Chemistry.

[CR157] Zhao Q, Stoyanova R, Du S, Sajda P, Brown TR (2006). HiRes—a tool for comprehensive assessment and interpretation of metabolomic data. Bioinformatics.

[CR158] Zhou B, Xiao JF, Tuli L, Ressom HW (2012). LC-MS-based metabolomics. Molecular Biosystems.

